# Intricate Structure–Function Relationships: The Case of the HtrA Family Proteins from Gram-Negative Bacteria

**DOI:** 10.3390/ijms252313182

**Published:** 2024-12-07

**Authors:** Urszula Zarzecka, Joanna Skorko-Glonek

**Affiliations:** Department of General and Medical Biochemistry, Faculty of Biology, University of Gdansk, Wita Stwosza 59, 80-308 Gdansk, Poland; urszula.zarzecka@ug.edu.pl

**Keywords:** HtrA, serine protease, Gram-negative bacteria, regulation of activity, allostery, oligomerization, PDZ domain, regulatory loops

## Abstract

Proteolytic enzymes play key roles in living organisms. Because of their potentially destructive action of degrading other proteins, their activity must be very tightly controlled. The evolutionarily conserved proteins of the HtrA family are an excellent example illustrating strategies for regulating enzymatic activity, enabling protease activation in response to an appropriate signal, and protecting against uncontrolled proteolysis. Because HtrA homologs play key roles in the virulence of many Gram-negative bacterial pathogens, they are subject to intense investigation as potential therapeutic targets. Model HtrA proteins from bacterium *Escherichia coli* are allosteric proteins with reasonably well-studied properties. Binding of appropriate ligands induces very large structural changes in these enzymes, including changes in the organization of the oligomer, which leads to the acquisition of the active conformation. Properly coordinated events occurring during the process of HtrA activation ensure proper functioning of HtrA and, consequently, ensure fitness of bacteria. The aim of this review is to present the current state of knowledge on the structure and function of the exemplary HtrA family proteins from Gram-negative bacteria, including human pathogens. Special emphasis is paid to strategies for regulating the activity of these enzymes.

## 1. Introduction

HtrA proteins are an evolutionarily conserved family of serine proteases present in both prokaryotes and eukaryotes. In humans, four HtrA homologs have been identified that are involved in important vital functions such as maintenance of cellular homeostasis, cell signaling and apoptosis. Altered levels of human HtrA often accompany cancer, arthritis, and neurodegenerative disorders [1,2,3].

Bacterial HtrAs are considered potentially important therapeutic targets for antimicrobial drug development. However, the creation of safe inhibitors that would be specific for bacterial homologs and would not inhibit the action of human HtrAs requires a thorough investigation of the three-dimensional (3D) structures and understanding of the mechanisms of action of these proteases. Structural studies of the HtrA proteins have been carried out for many years. The first HtrA protein with a solved 3D structure was the substrate-free DegP of the Gram-negative bacterium *Escherichia coli* [4]; later, structures in the presence of substrate [5,6] and structures of homologs from other organisms (other bacterial species, and plant and human homologs) were characterized [7,8] (summarized in Table 1). This allowed the identification of common features within the HtrA family of proteins, as well as fundamental differences specific to an individual homolog.

Proteases of the HtrA family are an excellent example illustrating how structural changes in the molecule modulate the activity of the enzyme and determine its proper function. A significant number of the HtrA homologs have been subjected to careful biochemical analysis, which showed that HtrA proteins exhibit allosteric properties and require the binding of appropriate allosteric modulators for activity. It is assumed that in the inactive form (resting state), the active centers of these proteins are inaccessible and/or are in the improper conformation to catalyze the reaction. Binding of an allosteric activator or a substrate molecule (acting as an allosteric activator) induces structural rearrangements in HtrA that make the active site available for the substrate and ensure the proper organization of the amino acid residues necessary for the peptide bond hydrolysis reaction [9].

In this work, we present an overview of commonly accepted activation models for two *E. coli* HtrA homologs, DegS and DegP, enriched with the latest literature data. We also describe the structure and function of selected HtrA homologs from Gram-negative pathogenic bacteria, focusing on differences from the model *E. coli* HtrAs.

**Table 1:** Selected 3D structures of bacterial HtrA homologues.

Organism	Protein	PDB Entry	Quaternary Structure	Substrate/ Peptide	Reference
*E. coli*	DegS	4RR1	Trimer	No	[10]
		4RQZ	Trimer	Yes	[10]
		1VCW	Trimer	Yes	[11]
*E. coli*	DegQ	3STI	Trimer	No	[12]
		3STJ	Trimer	No	[12]
		4A8A	12-mer	Yes	[13]
		4A9G	24-mer	Yes	[13]
*E. coli*	DegP	1KY9	Hexamer	No	[4]
		2ZLE	12-mer	Yes	[5]
		3CS0	24-mer	Yes	[5]
		3MH4	Hexamer	No	[14]
		3MH7	24-mer	Yes	[14]
		3OTP	24-mer	Yes	[15]
		3OU0	24-mer	Yes	[15]
		4A8D	12-mer	Yes	[13]
*H. pylori*	HtrA	5Y28	Trimer	No	[16]
	HtrA	7XS2	Monomer	No	[17]
*C. jejuni*	HtrA	6Z05	12-mer	No	[18]

## 2. Functions of Bacterial HtrAs

**Figure 1:**
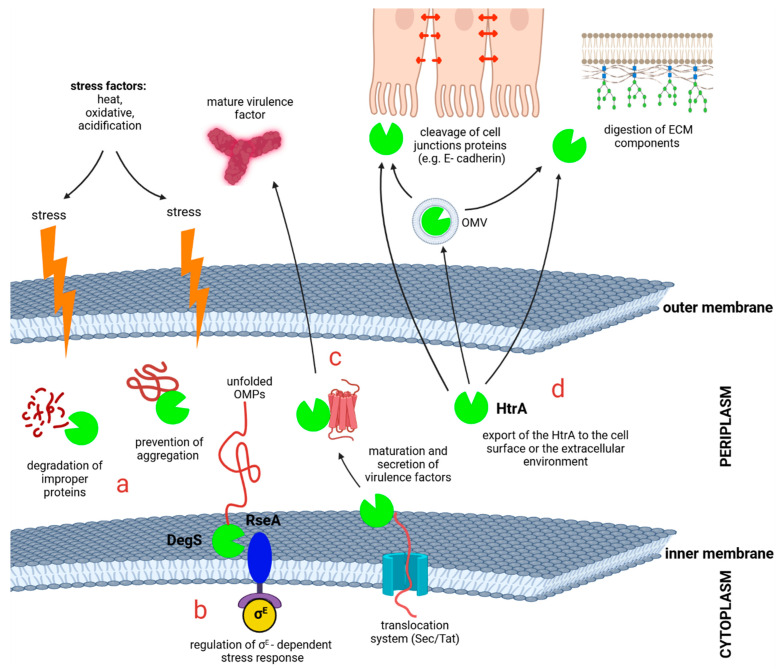
The role of the HtrA homologs in Gram-negative pathogenic bacteria. HtrAs are important in the various processes that are crucial for virulence and counteract the consequences of stress conditions. (**a**) HtrAs can function as protein quality control system components by digesting improperly folded proteins and preventing their aggregation. (**b**) Transcription of the σ^E^-dependent genes requires digestion of the anti-sigma factor RseA. In response to the presence of unfolded OMPs, DegS becomes activated and cleaves RseA, and these events finally lead to the release of the transcriptional factor σ^E^. (**c**) HtrAs are involved in secretion and proper maturation of several virulence factors. (**d**) HtrAs can be secreted outside the bacterial cell (on OMVs or by another hitherto unrecognized route). In the extracellular space, HtrAs act as virulence factors by cleavage of the cell junction proteins (E-cadherin, occludin, and claudin), or/and digestion of the extracellular matrix (ECM) components.

In the Gram-negative bacteria, HtrAs are soluble or membrane-bound proteins, localized to the cellular envelope or secreted out of the cell, that perform a variety of functions [19] (Figure 1). Most of the known HtrA homologs are involved in housekeeping functions. HtrAs are very important elements of the extracytoplasmic protein quality control system. Their task is to degrade the irreversibly damaged proteins from the cellular envelope and prevent protein aggregation under stressful conditions. The functions of HtrAs do not have to be only destructive, as it was proposed that some of the homologs are also involved in the outer membrane biogenesis and export/maturation of virulence factors [1]. For example, *Bordetella bronchiseptica* HtrA is required for correct processing and release of a key virulence factor, filamentous hemagglutinin (FhaB) [20]. A second very important function performed by the HtrA family is participation in the regulation of the stress response. Regulatory HtrAs are anchored to the cytoplasmic membrane by a trans-membrane segment. This location enables participation in the signal transduction from the cellular envelope to the cytoplasm. For example, DegS of *E. coli* co-localizes with its substrate, the anti-sigma factor RseA, in the cytoplasmic membrane, and it participates in activating the σ^E^ (σ^24^) factor-dependent extracytoplasmic stress response system. In response to the presence of misfolded outer membrane proteins (OMPs) in the periplasm, DegS starts a degradation cascade leading to destruction of the anti-sigma factor RseA. This leads to the release of the σ^E^ subunit and allows it to bind to the core of RNA polymerase [21].

A recently discovered role of the HtrA proteins is their direct involvement in the pathogenesis of bacterial infections as secreted virulence factors. The mode of secretion is not fully understood, but HtrA has been detected in the outer membrane vesicles (OMVs) (reviewed in [22,23]). The flagship example is the HtrA protein of *Helicobacter pylori*. It has been shown that the extracellular fraction of HtrA cuts certain proteins of gastric epithelial cell junctions and thus enables paracellular transmigration of bacteria across the epithelium. This process is extremely important for *H. pylori*, since the injection of the virulence factor CagA (Cytotoxin-associated gene A) takes place on the basolateral side of the epithelium. Once inside the host cell, CagA undergoes phosphorylation and affects host cell signaling, leading eventually to malignant transformation [24]. The HtrA homologs of other bacteria (e.g., *Borrelia burgdorferi*, *Campylobacter jejuni*, and *Chlamydia* sp.) can also function as secreted virulence factors and they may participate in dissemination of the pathogen in the host (reviewed in [22]). In addition to cleavage of intercellular junctions, they can degrade the extracellular matrix proteins (e.g., *B. burdorferi* HtrA) [25]. The latter functions indicate that the extracellular HtrA proteins may become attractive therapeutic targets for the development of low-molecular-weight inhibitors and/or anti-HtrA immune response strategies.

## 3. Structural Features of the HtrA Proteins

### 3.1. General Information on the Structure of HtrA Proteases

**Figure 2:**
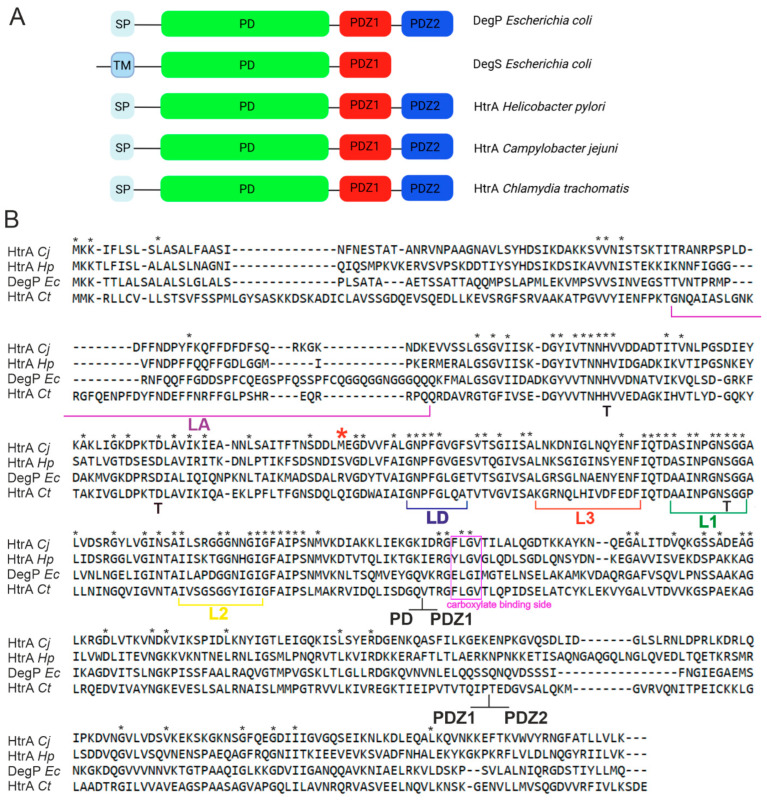
Comparison of (**A**) domain organization and (**B**) amino acid sequences of the selected HtrA homologs from Gram-negative bacteria. The amino acid sequences of HtrA *C. jejuni* (Q0P928), HtrA *H. pylori* (G2J5T2), DegP/HtrA *E. coli* (P0C0V0), and HtrA *C. trachomatis* (A0A0H2X3D0) were compared using PROMALS3D alignment [27]. The important regulatory loops (LA, LD, L1, L2, L3) and domain organization are marked. PD stands for the protease domain, T—catalytic triad, TM—transmembrane region, SP—signal peptide. Black asterisks indicate the conserved residues. The red asterisk indicates position of the 170 residue in HtrA*_Hp_* (position of naturally occurring Ser/Leu substitution, affecting the stability of the trimer).

According to the MEROPS database, HtrAs belong to clan PA, family S01 (with chymotrypsin α as the clan and family type protease), subfamily S1C of peptidases [26]. HtrA proteins are distinguished by the characteristic organization of their molecule, in which the chymotrypsin-type proteolytic domain occupies the middle position. At the C-terminus there is at least one PDZ domain (where PDZ stands for postsynaptic density protein 95 kDa, Disc large and zonula occludens protein 1), while the N-terminal region is highly variable and contains an export-directing signal peptide or transmembrane sequence to anchor the protein to the membrane, or possibly regulatory sequences (Figure 2). The individual domains are connected by flexible linkers that provide the molecule with high plasticity [7,14].

**Figure 3:**
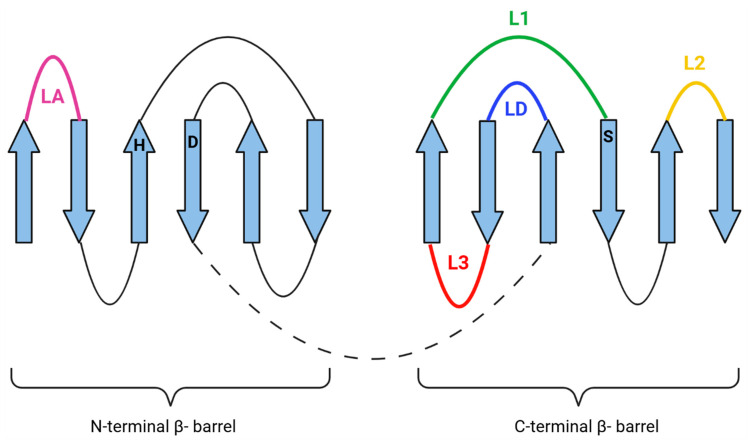
Schematic illustration of the main secondary structures within the proteolytic domain. β-strands are depicted as broad arrows. α-helical structures have been omitted for clarity (modified from [22]). The regulatory loops L1, L2, L3, LA, and LD are indicated. The active side triad residues Ser, His, and Asp are shown as S, H, and D, respectively. The loop connecting N- and C-terminal β-barrels is shown as a black dashed line.

Most of the knowledge about the structure and function of bacterial HtrA proteases comes from studies of homologs from bacterium *E. coli*: DegP and DegS. The active center of the HtrA protease is located in the gap between two six-stranded β-barrels building the proteolytic domain. The mobile loops connecting the individual β-sheets, LA, LD, L1, L2, and L3 (termed according to chymotrypsin nomenclature), play extremely important roles in catalyzing the reaction and regulating activity (Figure 3) [9]. Changes in the spatial arrangement of these loops allow switching between the active and inactive forms of the protease (discussed below).

The C-terminal PDZ domains are the modules responsible for protein–protein interactions, substrate binding, and activity regulation. The PDZ domain contains a cleft with a highly conserved carboxylate-binding loop (the Arg/Lys-XXX-Gly-Φ-Gly-Φ motif, where X is any amino acid residue and Φ is a hydrophobic residue) which is responsible for recognition and binding of peptide ligands [9,28].

**Figure 4:**
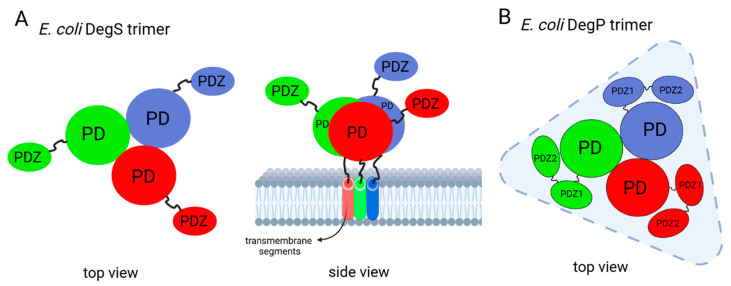
Schematic representations of the *E. coli* (**A**) DegS and (**B**) DegP trimeric units. Scheme of the DegS trimer is based on [11], while that of the DegP trimer is based on [1].

All HtrA proteins characterized so far are homo-oligomers. The basic building and functional unit is a trimer, which is maintained by interactions between centrally located proteolytic domains. The PDZ domains are located at the periphery of the trimer (Figure 4) [4,7].

### 3.2. One Building Unit, Many Combinations

Solving the native structures of HtrA proteins is challenging due to their large size and significant diversity and plasticity. In the case of several HtrA homologs, trimers can assemble to form higher-order structures: hexamers, 12-mers, 18-mers, 24-mers, and others [7]. The organization of oligomers depends on the presence/absence of allosteric modulator molecules, interactions with cell membranes, temperature, and the concentration of the protease itself [7,29].

**Figure 5:**
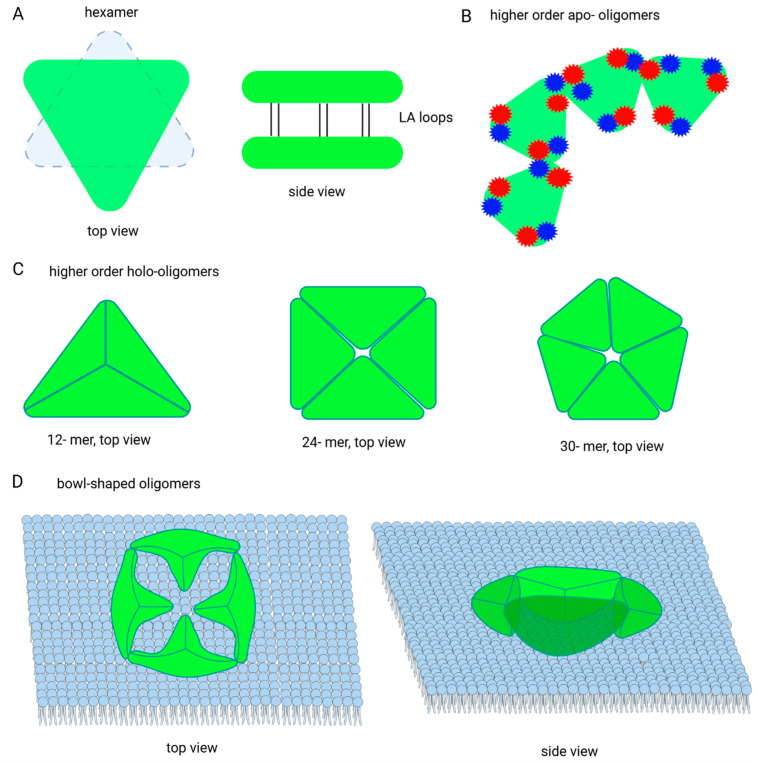
Schematic representation of the *E. coli* DegP higher-order oligomers. (**A**) Hexamer. (**B**) Higher-order apo-oligomers according to [29]; blue and red stars indicate position of the PDZ1 and PDZ2 domains. As can be seen, not all PDZ domains are engaged in the inter-trimer interactions in these assemblies. (**C**) Higher-order holo-oligomers according to [30]; all PDZ domains participate in the inter-trimer connections (not shown for the clarity of the schemes). (**D**) Bowl-shaped oligomers according to [31].

The diversity of oligomeric structures has been very well studied on the model of DegP from *E. coli* (Figure 5). The X-ray crystallography provided the first data on the 3D structure of DegP in the absence of substrate (an “apo” resting form) [4] and in the presence of ligand molecules (a “holo” form) [5,15].

The “apo” form of DegP is a hexamer, formed by a staggered association of two trimers (Figure 5A) [4]. This structure is maintained by interactions between the PDZ1 and PDZ1′ domains (prime denotes that the structure does not belong to the same protomer) of the opposing trimer, as well as via components of the proteolytic domains—elongated LA loops originating from opposite subunits (Figure 5B). The proposed spatial model of the LA loops in the hexamer suggests formation of a hydrophobic cluster by these structures, which can stabilize the hexamer [32]. In this “apo” conformation, the two trimeric units are arranged in a way that restricts access to the catalytic centers. In the hexamer, the catalytic centers are hidden inside a central compartment, access to which is blocked by two PDZ domains that form “side walls”. In addition, the LA loops from the subunits of one trimer form inhibitory contacts with the L1 and L2 regulatory loops in the subunits of the opposing trimer, directly covering access to the active center [4].

In the presence of substrate molecules or allosteric activators, DegP forms spherical structures composed of 4, 8, or other numbers of trimers. In the central part of such a spherical oligomer there is a chamber in which the substrate can be encapsulated; hence, the name “cage” oligomer is often used [5,6]. Spherical structures are maintained via interactions between the PDZ domains. Specifically, the PDZ1 domain of one trimer interacts with the PDZ2′ domain of the other trimer.

A widely accepted model of DegP activation based on crystal structures of the protease in the absence and in the presence of substrates, as well as a range of experimental data, assumes the following transformations of DegP oligomers. In the absence of appropriate substrates in the cell, the protease is present as an inactive hexamer. Under stress conditions and/or substrate abundance, the hexamer dissociates into trimers that can subsequently form a cage-like substrate containing structures [6,26].

Recent studies have shed new light on the DegP conformation landscape in solution under conditions mimicking the natural setting of the periplasm (including physiological concentrations of DegP in the range of 40–200 µM and ionic strength of 200–300 mOsm/L). It was shown that hexameric structures predominate only at low, non-physiological temperatures for *E. coli* (5–20 °C), while at higher temperatures (20–50 °C), depending on the concentration of DegP, dissociation of hexamers to trimers and possibly formation of higher-order oligomers occurs. In particular, DegP of a concentration of 10 µM occurs mainly in the form of trimers. Higher concentrations of DegP (>30 µM) at temperatures of 30–40 °C already allow the formation of higher-order oligomers. A further increase in temperature (40–50 °C) leads to re-dissociation of these assemblies [29,33]. However, the oligomeric structures formed at elevated temperatures are not the same as the caged holo-oligomers formed in the presence of substrate. Apo higher-order oligomers (substrate-free oligomers) are also maintained through PDZ1-PDZ2′ interactions, but in these structures not all PDZ1 and PDZ2 domains are involved in inter-trimeric interactions; thus, they represent partly structured assemblies (Figure 5B). It is important to note that in the cage holo-oligomers, a complete set of possible PDZ1-PDZ2′ interactions is formed. Therefore, the apo higher-order oligomers are rather unstable, rapid exchange of trimeric subunits is likely to occur, and they probably represent a kind of network of preorganized assemblies, ready to capture clients and associate into cage holo-oligomers [29]. Relationships between the apo structures, as well as possible pathways and mechanisms of DegP activation, are further discussed in the following sections.

In a recent paper [30], it was shown that the size of the *E. coli* DegP oligomer is determined by the size of the bound substrate. Using substrate molecules of sufficiently large size, it was shown that DegP can form very large cage-like structures, composed of up to 60 subunits. These assemblies can be treated as polyhedrons with triangular sidewalls (corresponding to trimers). The corners of these structures are formed by inter-trimeric interactions of PDZ1-PDZ2′ domains (Figure 5C). The ability to form such structures that differ significantly in surface curvature is due to the presence of flexible linkers between the proteolytic domain and PDZ1 and between PDZ1 and PDZ2. The capacity to assemble into oligomers of various volumes, depending on the size of the substrate molecules, is a very valuable property of DegP. As a housekeeping protein, DegP interacts with many cellular clients, sometimes very large ones. Therefore, the ability to form appropriately tailored cages enables it to function efficiently under stress conditions when a wide range of potential substrates for DegP accumulate.

DegP also binds to lipid membrane structures, and in this case the oligomer assumes the shape of an open hemisphere—a “bowl”, composed of several (3–5) trimers (Figure 5D). In bowl-shaped oligomers, the trimeric subunits bind via the same interactions that stabilize the other described higher-order structures, i.e., via PDZ1-PDZ2′ contacts at the trimer interface. As in the case of the apo higher-order oligomers, these interactions are less stable than those in the cage-like holo oligomers. This property ensures high plasticity of the bowl-shaped structures. The catalytic centers are located inside the bowl and appear to be well accessible to substrate molecules [31]. The formation of bowl-shaped oligomers appears to stimulate proteolytic activity, as DegP bound to liposomes degrades model substrates much more efficiently [31,34].

## 4. Molecular Aspects of HtrA Functioning

In the bacterial HtrA proteins biochemically characterized so far, there is an allosteric regulation of the proteolytic activity, based on signal transduction from the PDZ domain to the proteolytic domain and between oligomer subunits. In the resting form, the lack of proteolytic activity can be due to the following reasons: (1) a non-functional active center and/or (2) difficult access or lack of access to the active center [9]. The PDZ domain contains a binding site for an allosteric modulator, which can be a peptide molecule of a specific amino acid sequence (which is not an HtrA substrate) or a fragment of a substrate molecule (also of an appropriate amino acid sequence) [9]. The mechanism of activation itself can vary between HtrA homologs and can be based on one of two main strategies: (1) relief of inhibition or (2) peptide-activation model [35].

The activation mechanisms of two *E. coli* HtrA homologs, DegS and DegP, have been most thoroughly investigated. Both proteins become activated by binding of appropriate peptide ligands to the PDZ domain. The common activating peptides are the C-terminal fragments of the outer membrane porins (OMPs) containing the Tyr-X-Phe motif, where X stands for any amino acid [36,37]. The C-terminal sequences of OMPs are hidden in the beta-barrel structure in properly folded proteins. Their exposure indicates protein misfolding and signals folding stress in the cellular envelope [38].

### 4.1. The Mechanism of E. coli DegS Activation

DegS is a protein anchored to the cytoplasmic membrane by an N-terminal segment. It contains only one PDZ domain, and its functional form is a trimer (it does not form higher-order oligomers). The shape of the molecule resembles a funnel open to the periplasm with the PDZ domains exposed on the outermost side (Figure 4A) [11]. DegS seems to follow the “relief of inhibition” mode of regulation, where the PDZ domain is an inhibitory module [35], and its removal causes an increase in protease activity [36,39]. Certain loops of the proteolytic domain play a key role in the activation and subsequent maintenance of the active structure: L1, L2, L3, and LD [10,11].

**Figure 6:**
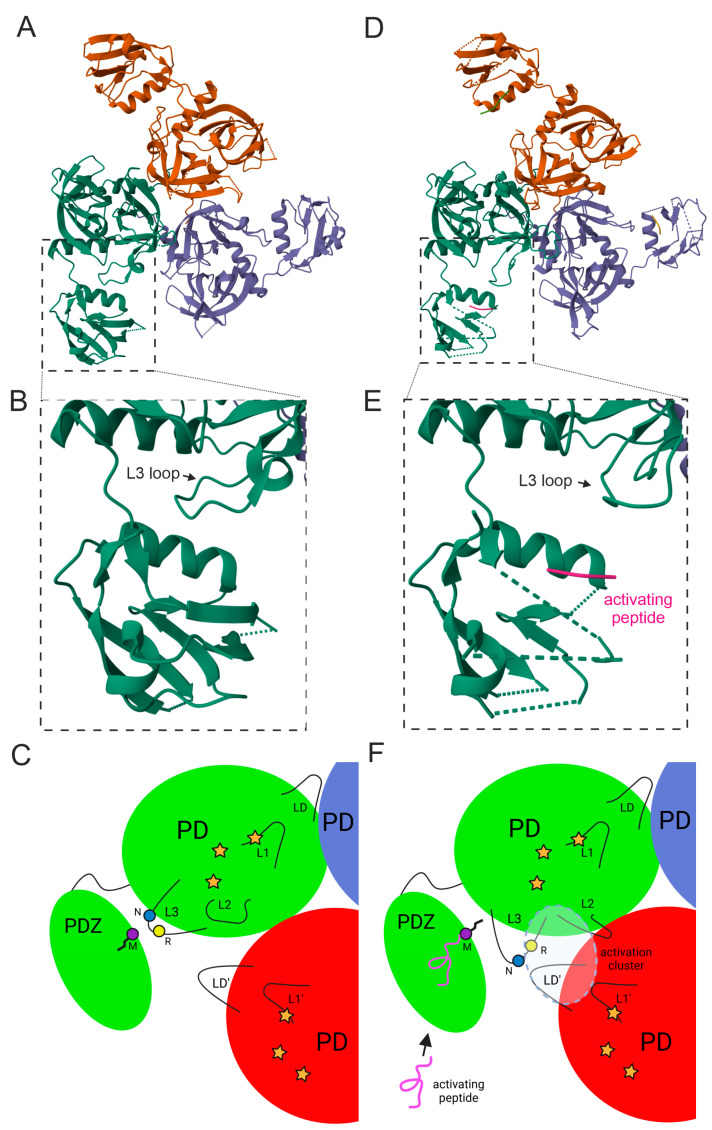
Allosteric activation of DegS. (**A**–**C**) The resting state of the protease based on PDB entry 4RR1, and (**D**–**F**) the active state with bound ligand (shown in magenta) based on PDB entry 4RQZ. Active site residues were shown as yellow stars. The schemes (**E**,**F**) are based on [22,35]. Activation cluster is shown as a dashed line.

Analysis of the crystal structures of DegS in its inactive form and in complex with the activation peptide indicates a possible mechanism for protease inhibition by PDZ. Interactions of the unliganded PDZ domain with the proteolytic domain are thought to stabilize the inactive conformation of DegS. Of particular note are salt bridges between acidic residues of the PDZ domain and basic residues of the proteolytic domain. In particular, Arg178 (L3 loop) interacts with Glu317 and Asp320 (PDZ domain), and Lys243 (proteolytic domain) with Glu324 (PDZ) [39,40,41]. Binding of the activating peptide to PDZ removes inhibitory contacts between the PDZ and proteolytic domains. Interaction of the peptide ligand with the PDZ binding cleft triggers a steric clash between the Met319 residue in the PDZ and the Asn182 residue in the L3 loop, which forces conformational changes within the L3 loop and its repositioning, leading to the breaking of autoinhibitory interactions and activation of the protease (Figure 6B,E). Such an activation mechanism, based on steric hindrance, is believed to be conserved in other DegS homologs in related bacterial species. It has been observed that in Gram-negative proteobacteria, DegS homologs contain Met or Leu residues at the position corresponding to 319, while the position corresponding to residue 182 is usually occupied by Ser or Asn [35].

The L3 loop (residues 176–189) plays a key role in DegS activation. In the inactive form of DegS, the L3 loop is involved in definitely autoinhibitory interactions. It is necessary to mention not only the above-mentioned interactions with the PDZ domain [39], but also contacts with the proteolytic domain, stabilizing its resting form. For example, the Leu181 residue (L3) sits in a hydrophobic pocket formed by non-polar C-terminal residues of the proteolytic domain. The L181A substitution causes an 80-fold increase in the basal activity of DegS [35]. On the other hand, the Arg178 residue (L3) is involved in stabilizing the active form of DegS as well (discussed below) [10]. Therefore, it can be concluded that L3 plays important roles in maintaining DegS in both inactive and active forms.

The mechanism of allosteric activation proposed in the paper [42] assumes that binding of an OMP peptide to one PDZ domain stimulates the activation of two proteolytic domains: one from a given subunit and one from an adjacent one (cis and trans activation). This phenomenon can be explained by the existence of a so-called “allosteric activation cluster” in the structure of the active protease [10]. This cluster includes amino acid residues from two adjacent subunits, which are located in the L3 loop (Arg 178) of one subunit and components of the proteolytic domain of the adjacent subunit, including the LD’ loop (Leu164, Tyr162, and Pro161 residues). A network of hydrogen interactions (side chains of Thr167, Arg178, and Gln191 and main chain atoms of amino acids Pro161, Leu164, Phe220, and Ile232) and hydrophobic interactions (side chains of Tyr162, Leu164, and Phe220, Ile232, and Phe243 of both subunits) is formed. Correct formation of this cluster enables the adoption and stabilization of the functional structure of the active center. In particular, the side chain and main chain atoms of Pro161 and Tyr162 form contacts that stabilize the proper active site loop L1 structure (res. 198–201), enabling the proper alignment of Ser, His, and Asp of the catalytic triad and leading to the formation of the oxyanion hole. Additionally, the side chains of Thr169 and Asn197 form hydrogen bonds that indirectly connect the active-site loop L1 to the components of the activation cluster.

In the inactive conformation, many components of the activation cluster occupy positions significantly different from those in the active conformation, including the Arg178, Phe220, and Tyr162 residues. This results in the loss of all stabilizing hydrogen bonds of the activation cluster, leading to an improper active-site loop structure and a lack of the oxyanion hole. According to the model proposed in the publication [10], based on the crystal structures of DegS from *E. coli* and the DegS homolog from *Mycobacterium tuberculosis* [43], the activation peptides bound in the PDZ domain do not contact the activation cluster. Moreover, although the PDZ-bound OMP peptide is adjacent to L3, no significant interactions between the peptide and L3 have been identified [40]. This observation strongly supports the hypothesis that ligand binding rather destabilizes autoinhibitory interactions and does not stabilize active conformation of DegS. Interestingly, binding of the substrate molecule (RseA protein) may play a stabilizing role in maintaining the active conformation of DegS, as it results in the fusion of the active center loop and activation cluster in the trimer [10].

Summing up, the activation signal is transmitted from the PDZ domain, through the L3 loop and then the LD’ loop to the active site. The signal transmission involves structural changes in the L3 and LD’ loops, which enables the formation of proper interactions with the L1′ and L2′ loops. The following events, depicted in Figure 6, take place:

1. In the absence of the ligand, the L3 loop forms autoinhibitory interactions with the PDZ domain.
2. Binding of the OMP peptide to the PDZ domain causes a steric clash with the amino acid side chain of the L3 loop.
3. The steric clash forces a spatial rearrangement of the L3 loop, which disrupts autoinhibitory interactions.
4. The change in the structure of the L3 loop results in the Arg178 residue gaining the ability to form new contacts with the neighboring subunit to proteolytic domain residues (including LD’ loop).
5. A network of hydrogen bonds and hydrophobic interactions (activation cluster) is formed, which stabilizes the active conformation of the protein and facilitates communication between subunits.
6. A properly formed network of interactions allows the active center loop to adopt the correct conformation and form the oxyanion hole.

### 4.2. Mechanism of DegP Activation

The DegP protease is characterized by a more complex regulatory mechanism. Among other factors, it is due to a different monomer composition, a more advanced oligomer structure, but also a different function performed by this protein. DegP is a housekeeping protease; thus, it should be able to degrade a big variety of substrates. Therefore, the regulatory strategy is based not only on turning on protease activity under conditions of substrate abundance, but also on restricting access to active centers for inappropriate substrates. The substrate selectivity of DegP is not particularly high. It is capable of degrading a wide range of proteins, provided that their structure is unnatural, at least partially unfolded and exposing hydrophobic amino acid residues. This protease preferably hydrolyses peptide bonds between paired hydrophobic residues [1]. Unlike DegS, DegP contains two PDZ domains and forms higher-order oligomers made up of different numbers of trimeric subunits. As discussed in Section 3.2, the resting form of the protease is a hexamer (at low temperatures) or a population of higher-order oligomers with fairly loose connections between trimeric subunits [29,33]. In the presence of substrate molecules (proteins or peptides), the interactions in the oligomer are altered and the hexamer or apo-oligomers are transformed into cage-like structures composed of four or more trimeric subunits [9,30].

**Figure 7:**
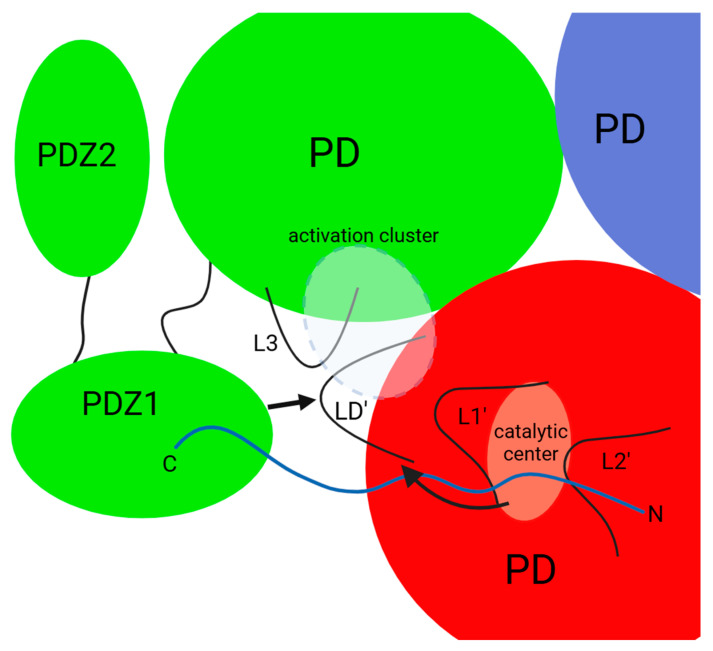
Schematic model of allosteric activation of DegP. The activating peptide/substrate (shown in blue) binds simultaneously to the PDZ1 domain of one protomer (green) and the active center of the neighboring protomer (red). The signals are transmitted from both binding sites and they presumably meet at the LD’ loop. The activation cluster (dashed line) is formed, leading to activation of the protease. Based on [10,22,45].

The process of allosteric activation of DegP is based on a “peptide activation” strategy and requires binding of the activating molecule (which is most often the substrate itself) simultaneously at two sites of the enzyme: (1) the N-terminal fragment in the catalytic center and (2) the C-terminal portion in the PDZ1′ domain of the adjacent subunit [15] (Figure 7). The PDZ1 domain is essential for proteolytic activity of DegP. In contrast to DegS, deletion of PDZ1 results in a lack of proteolytic activity [44]. In DegP, also an activation cluster is formed, analogous to that found in DegS, in which a key role in the formation of the hydrogen bond network is attributed to the side chains of the Thr176, Ser183, Arg187, and Gln200 residues [33,35]. In the inactive conformation, these residues do not form contacts [4,15,37]. Replacing any of these residues with amino acids incapable of participating in the activation cluster interaction network significantly reduces DegP’s ability to degrade substrates. The presence of an activation cluster with conserved key residues suggested the occurrence of a DegS-like activation mechanism with the direction of signal transduction from the PDZ toward the proteolytic domain. However, results showing that binding of the peptide exclusively at the active site also leads to DegP activation (albeit markedly weaker than binding in the two positions of DegP) suggest that the activation mechanism is more complicated.

Based on analyses of the available 3D structures of DegP in the resting and active forms, and the results of a series of experimental works, an alternative allosteric signaling pathway has been proposed (Figure 7). According to it, binding of an allosteric ligand at two sites, i.e., the PDZ1 domain and the active site of the neighboring protomer, causes simultaneous transmission of a signal from both locations. The two signals are likely to meet at the LD’ loop, which in DegP is a relatively static element (unlike in DegS) and does not change its position throughout the activation process. It is therefore possible that the LD’ loop serves as a kind of scaffold at the subunit interface for other interacting loops: L3, L1′, and L2′ [45]). How the L1 and L2 loops are released from the inhibitory interactions with the LA’ loop and the hexamer is dissociated is not known. The LA loops are flexible, and a significant portion of these structures has not been visualized by crystallographic or cryo-EM studies. Consequently, it is difficult to propose a reliable model based on the known structures of DegP. On the other hand, DegP hexamers are not the dominant oligomeric form at temperatures above 30 °C (i.e., physiological conditions for *E. coli*), but rather higher-order apo-oligomers [29]. Therefore, issues related to inactive hexamer disassembly may not be of physiological significance.

Recently published cryo-EM structures of DegP 12-mer in complex with a model substrate molecule (human telomere repeat binding factor; hTRF1) has allowed visualization of DegP–substrate interactions [30]. The structure clearly shows the C-terminal fragment of the substrate, bound to the enzyme at two sites: the PDZ1 domain of one protomer and the protease domain of the adjacent protomer within the same trimer, which is consistent with previous reports [14,15]. An in-depth analysis of this complex led to the detection of substrate–PDZ1 interactions that appear to stabilize PDZ1-PDZ2′ inter-trimeric connections through the formed substrate–PDZ1-PDZ2′ cage interface. In particular, substrate amino acid side chains contribute to the crucial interdomain hydrophobic interface, involving Leu276, Met280, Phe289 (PDZ1), and Tyr444′, Leu446′ (PDZ2′). A number of contacts between the substrate and the neighboring subunit protease domain were also identified. Interestingly, the substrate segments bound to the proteolytic domain have a characteristic V-shape, which is achieved by formation of two non-native β-strands at the expense of a native α-helix. The consequence of the formation of such a bent conformation is that the substrate adopts the correct orientation in relation to the active site serine, with the cleavage site at the base of the “V” [30].

The formation of the 12-mer cage oligomer upon hTRF1 binding seems to be strongly cooperative, as it can be inferred from the lack of a significant number of intermediates in the formation of 12-mers. The appearance of 12-mers was observed already at low substrate concentrations, and the complete transformation of the DegP pool into a 12-mer occurs already for a 2:1 DegP:substrate ratio, i.e., without saturation of all substrate binding sites, which emphasizes the allosteric communication between trimeric subunits. Furthermore, obtaining an active conformation by the trimeric subunit requires a coordinated transition of all protomers to the active form [33]. The authors propose that such a property may explain the functioning of DegP as a protease and as a chaperone. In the absence of complete saturation/activation of protomers, the client proteins bound in the cage would not undergo proteolysis, being simultaneously isolated from the external environment.

### 4.3. Temperature-Dependent Regulation of the DegP Protease

*E. coli* DegP is a protein whose activity is significantly regulated by temperature. At low temperatures (below 20 °C), substrate hydrolysis is very slow, whereas an increase in temperature above the optimal for *E. coli* (37 °C) causes a rapid increase in the rate of substrate digestion [46]. What structural changes are induced by an increase in temperature? Early studies on thermal activation of DegP showed that an increase in temperature above 37 °C causes a transition from the hexameric to the trimeric form [5,6]. Moreover, as the temperature increases, the regulatory loops LA, L2, and L1 become gradually exposed to the solvent, which indicates structural changes occurring in or around these loops [47]. Recently published data shed more light on this process and provided more details on the mechanism of thermal activation of DegP. Biophysical analysis of the DegP molecules, using Fourier transform infrared spectroscopy and temperature-jump nanosecond time-resolved IR difference absorbance spectroscopy, revealed that the temperature change is sensed at the trimer–trimer interface and the unfolding/disassembly process is propagated sequentially, eventually leading to the dissociation of the hexamer [48]. Further information was provided by detailed structural studies of DegP performed using high-resolution nuclear magnetic resonance (NMR) spectroscopy [49]. The authors proposed that an interdomain lock, composed of Met280 (PDZ1) and Tyr444 (PDZ2′), is present at the trimer–trimer interface and acts as a temperature sensor to control oligomer rearrangements. This hypothesis is quite surprising, since in the crystal structure of the DegP hexamer (PDB ID: 1KY9) these interactions were not visualized; the arrangement of the PDZ domains at the trimer interface is different and indicates PDZ1-PDZ1′ interactions. However, previous experimental reports have already indicated an important role of the Tyr444 residue in maintaining the hexamer structure. Mutant DegP Y444A variants occur in a trimeric form at 25 °C and are also characterized by increased proteolytic activity [50]. The M280A substitution also destabilizes the hexamer and increases DegP activity [49]. The analyses carried out by the authors demonstrate that temperature rise causes the disruption of Met280-Tyr444 interactions, as well as increases the dynamics of the molecule. The key role in the temperature-dependent modulation of the interdomain lock action is attributed to the Met280 residue. Under heat shock conditions, Met280 gains high flexibility. As the dynamics of the core and side chains of amino acids in PDZ1 increases simultaneously, the interaction with Tyr444 is broken and the PDZ1 domain is liberated. Again, it has not been clearly explained how the active site loops are released from interactions with the LA loops during hexamer dissociation. It has been proposed that during signal transduction from the PDZ1 domain, tensions are generated in the main chains of the LA loop, which may consequently lead to detachment of the LA loop from the opposing subunit [48]. Increased exposure to the environment/mobility of the LA loop during thermal activation has also been proposed based on biophysical studies [47] and in silico [51].

Very interesting information was provided by studies on the temperature-dependent DegP oligomerization pathways [29]. It has been shown that in the absence of a substrate, DegP occurs as a dynamically changing ensemble of complexes composed of trimeric subunits. Two alternative pathways have been proposed that result in the association of DegP trimers: (A) leading to the formation of canonical hexamers (described by the crystal structure 1KY9 [4]) and prevailing at low temperatures, and (B) leading to the formation of labile higher-order apo-oligomers and occurring at physiological temperatures for *E. coli* (30–40 °C). As already mentioned, in apo-oligomers, not all PDZ1 and PDZ2 domains are involved in inter-trimeric interactions. This means that the latter structures are less stable and more plastic. Structural flexibility is crucial for substrate binding to occur via “induced fit” or “conformational selection,” which enables a faster response of DegP apo-oligomers to the appearance of misfolded proteins in the cell than would be the case for canonical hexamers [30]. Since the formation of apo-oligomers occurs in the physiological temperature range for bacteria, it is advisable to revise, at least in part, the classical mechanism of activation, in which an inactive hexamer is transformed into active cage oligomers. It should be also noted that the conversion of higher-order apo-oligomers into cages does not require the disruption of LA contacts with L1 and L2 active site loops, since these interactions are no longer present in these structures.

### 4.4. Oligomer Rearrangements and DegP Proteolytic Activity

During DegP activation, structural changes occur in the enzyme molecules and this process is accompanied by the formation of cage oligomers. However, it turned out that obtaining a caged structure is not essential for DegP proteolytic activity. DegP mutant variants incapable of assembling into higher-order oligomers (e.g., DegP Y444A) are fully active toward the substrates tested and show cooperative interactions with substrates in vitro [50].

So what is the significance of assembling into caged oligomers? Some clarification was provided by the results of studies using mutant DegP variants with impaired higher-order oligomer formation (Y444A, L276A, or L446A) in combination with a mutation stabilizing the active conformation (R207P) [52]. The presence of mutations that limit the formation of higher-order DegP oligomers does not significantly affect the growth of bacterial cells even under heat shock conditions; however, in competition tests at 45 °C, the *E. coli degP Y444A* strain is eliminated by wt *E. coli*, indicating reduced fitness of the mutant. The substitution of Arg207 for Pro results in a constant maintaining of the active conformation by the oxyanion hole. The DegP R207P mutein shows increased basal proteolytic activity, a reduced Michaelis constant, and tighter substrate binding compared to the wild-type protein. Moreover, the R207P mutation allows the formation of caged DegP oligomers at lower substrate concentrations than required for wt DegP. Expression of DegP R207P does not cause any visible growth disturbances in non-stressful conditions, but at elevated temperature (45 °C) distinct cell filamentation is observed. In contrast, the combination of two mutations, R207P and a mutation that disrupts the formation of caged oligomers (e.g., R207A/Y444A, R207A/L276A, or R207A/L446A combinations), is lethal under heat shock conditions (45 °C) or upon elevated expression of the mutant DegP variants. The above results allow the conclusion that allosteric regulation along with the formation of caged oligomers jointly provides the cell with an adequate level of proteolytic activity and proper access to DegP catalytic centers, restricting the repertoire of digested substrates. The formation of caged oligomers makes the catalytic centers accessible only to encapsulated substrates. In the case of cage-defective mutants, the active sites are constantly exposed; thus, selection of substrates is unrestricted. So, cage assembly is expected to provide an additional level of regulation of proteolysis, independent of allosteric control [52].

## 5. Structural and Functional Studies of HtrA Homologs in Pathogenic Gram-Negative Bacteria

Due to the participation of HtrA homologs in the pathogenesis of bacterial infections, these proteins are the subject of intensive studies, including structural studies. Among Gram-negative pathogens, the 3D structures of HtrAs from *H. pylori* and *C. jejuni* have been examined best. Both bacterial species are classified in Campylobacterota (previously Epsilonproteobacteria), a phylum evolutionarily distant from the other proteobacterial classes [53,54]. There are also literature reports on the HtrA structure of *Chlamydia trachomatis*. Many similarities in the structures of these HtrA homologs to that of *E. coli* DegP can be seen. First of all, these proteins are able to form higher-order oligomers in the presence of substrate proteins or relevant peptides, which may suggest allosteric regulation of their proteolytic activity. For example, *C. trachomatis* HtrA is expected to exhibit a mechanism of activation similar to that of *E. coli* DegP, relying on PDZ1-L3-LD’ interactions [14,55]. Similarities in the amino acid sequences (Figure 2) and structures of the proteolytic domains are also high. However, these proteins also show some features that distinguish them from model HtrAs from *E. coli*, indicating that the design of antimicrobial molecules cannot be based uncritically on model HtrA structures.

Below we present the structural characteristics of HtrA homologs from these selected bacterial species, with an emphasis on their unique features.

### 5.1. Chlamydia HtrAs

*Chlamydia* are obligate bacterial pathogens that are able to replicate exclusively in host cells. The genus includes several pathogenic species, including human pathogens *C. trachomatis* and *C. pneumoniae*, that cause ocular and genital infections or respiratory disease, respectively [56]. *Chlamydia* HtrA plays important roles over the entire developmental cycle of the pathogen. This protein seems to be necessary for bacterial survival, as the anti-HtrA inhibitor treatment blocks development of *Chlamydia* [57].

The resting form of *Chlamydia* HtrA is presumably a hexamer. In the presence of substrate molecules or activating peptides the higher-order oligomers are formed [58]. Unlike *E. coli* DegP, regardless of the size of the substrate (protein or peptide), the molecules of *C. trachomatis* HtrA assemble preferentially in 24-mers [58]. Chlamydial HtrAs are expected to associate with membranes [59,60]. Therefore, they are expected to adopt bowl-shaped structures as well. This assumption is supported by the analysis of surface epitopes of this protein. Basing on the structural models, it was demonstrated that the HtrA epitopes recognized by neutralizing anti-HtrA antibodies are exposed in the bowl structures but hidden in the spheric oligomers [59]. Thus, the HtrA molecules exposed on the OMVs shed by *Chlamydia* or on the surface of elementary bodies most probably are bowl-shaped structures.

Similarly to *E. coli* DegP, *C. trachomatis* HtrA is activated allosterically by PDZ1-binding peptide [58]. It has been shown that linkage between the PDZ1 domain and the L3 loop, as well as between the L3 loop and LD’, is required for proteolytic activity to occur. This indicates that the activation mechanism is similar to that occurring in *E. coli* DegP. However, the need for other contacts, not previously found in *E. coli* DegP, was observed. Specifically, the PDZ1 domain interactions with the protease loop LC’ and strand β5′ were required for efficient proteolytic activity. Substitutions R299L (PDZ1), which disrupts bonding with Asp169 in LC, and K160V (β5′), breaking contacts with Asp310 and Glu312 of PDZ1, markedly reduce substrate cleavage rates [55]. This finding may indicate existence of subtle differences in the mechanism of signal transmission from the PDZ domain or a distinct way to stabilize the enzyme’s active structure.

### 5.2. Campylobacter jejuni HtrA

*C. jejuni* is a pathogen which, in humans, causes gastroenteritis termed campylobacteriosis. This zoonotic disease is transmitted mainly from chickens, but also from cattle, pigs, and sheep [61]. Infection occurs through contact with infected meat, water, milk, and dairy products [62].

The *C. jejuni* HtrA protein is involved in the bacterial infection at the stage of adherence and invasion of human epithelial cells; the strains lacking this protease are impaired in both processes [63,64]. Transmigration of bacteria across the epithelial monolayer is also disabled due to a lack of HtrA proteolytic activity. The latter phenomenon is most probably related to the ability of a secreted fraction of HtrA to cleave components of intercellular junctions, such as E-cadherin, occludin, or claudin-8 [64,65,66].

*C. jejuni* HtrA can form various oligomeric forms. As demonstrated by blue native PAGE of *C. jejuni* cellular lysates, the wt HtrA is separated as a mixture of trimers, hexamers, and 12-mers, while the proteolytically inactive variant HtrA S197A exists only as large oligomers having a size exceeding 12-mer [67]. Results obtained using an alternative method, size exclusion chromatography (SEC), largely confirmed the above data. In the absence of substrates, purified preparation of the active protease was eluted at the position corresponding to hexamer, while HtrA S197A was a mixture of 12-mers and large oligomers. Addition of substrate molecules (β-casein) caused a shift to high-order oligomers in the case of HtrA S197A, while no change in the elution profile was observed for the wt HtrA [18]. Most probably, the substrate was degraded in the course of separation and the protease has returned to its resting state. Presence of 12-mers in the inactive protease preparation was explained by the presence of HtrA degradation products, which co-purified with the full-length protein even under denaturing conditions and most probably served as activation ligands. A similar phenomenon was observed in the case of the HtrA homolog from *Legionella* [68]. Based on the above results, it can be speculated that the resting form of *C. jejuni* HtrA is a hexamer. Moreover, in the presence of the substrate, higher-order oligomers are formed, the dimensions of which depend on the size of the substrate. This resembles the properties of *E. coli* HtrA. The 12-mer of *C. jejuni* HtrA, however, shows some features that distinguish it from the 12-mers of other characterized HtrAs (*E. coli* HtrAs and *Legionella* HtrA). The molecular dynamic simulation has shown that dodecamer is rather unstable and the contacts between trimers are loose, indicating that the 12-mers are flexible structures and can be easily rearranged depending on external conditions. These differences may be due to the slightly different positioning of PDZ2 domains compared to these in *E. coli* DegP, resulting in weaker inter-trimer PDZ1-PDZ2′ contacts [18].

### 5.3. Helicobacter Pylori HtrA

*H. pylori* is a human pathogen that colonizes the stomach and duodenum in more than half of the human population [69] and is a major risk factor for gastric cancer [70]. This type of cancer is the sixth most commonly diagnosed cancer and the fourth leading cause of cancer deaths [71].

In *H. pylori*, HtrA is one of the main virulence factors. Secreted HtrA is involved in the degradation of components of the intercellular adhesions, in particular adherens (E-cadherin), tight junctions (occludin and claudin-8), and desmosomes (desmoglein-2). This ability enables paracellular transmigration of the bacteria across the gastric epithelium [23,72] and subsequent injection of CagA effector into the host cells [73]. Due to the key role of HtrA in *H. pylori* virulence and its essentiality for bacterial growth, the protein has been intensively studied in terms of both functional and structural aspects.

The most likely resting oligomeric form of *H. pylori* HtrA is trimer. This structure of substrate-free HtrA was found to be dominant in experiments using size exclusion chromatography and sedimentation velocity ultracentrifugation. At higher concentrations, the protein seems to assemble into larger oligomers: hexamers, nonamers, and small quantities of 12- and 18-mers (HtrA from the *H. pylori* 26695 strain) or mainly 18-mers (HtrA from the *H. pylori* N6 strain). In the presence of a substrate (β-casein), *H. pylori* HtrA forms higher-order large oligomers of undetermined size [74].

**Figure 8:**
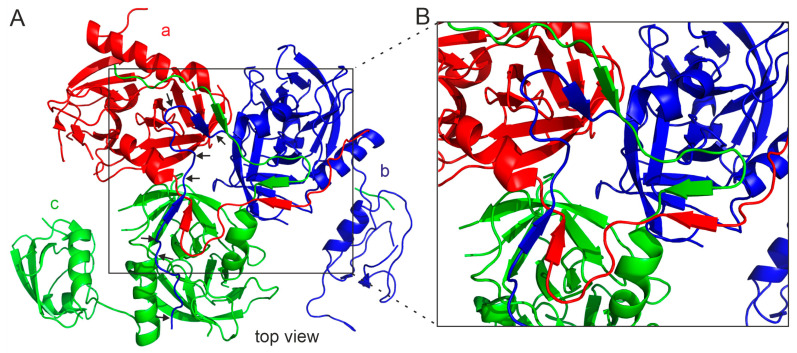
The domain swapping in the HtrA*_Hp_* trimer. (**A**) Individual subunits a, b, and c are marked in red, blue, and green, respectively. The black arrows indicate the N-terminal region of the “b” subunit, which penetrates the “a” and “c” subunits. (**B**) Enlargement of the area where domain swapping occurs. Based on PDB entry 5Y28. The protein structure images were generated using PyMOL version 1.3.

To date, crystal structures of *H. pylori* HtrA spanning the full length of the protein have not been determined. However, structures of HtrA lacking the PDZ2 domain [16] and HtrA without the N-terminal fragment (covering residues 43–475) are available [17]. In addition, a 3D structure has been obtained using the cryo-EM technique [16]. Analysis of these structures revealed very interesting structural features not previously observed in other HtrA proteins. The most notable of these is the occurrence of domain swapping at the N-terminal parts of the trimer subunits. Specifically, the N-terminal segments traverse the interface to two neighboring monomers. The extended N-terminus is mainly composed of two short β-strands and two loops: loop1 (Gln 21-Lys 29), β1 (Glu 30-Val 32), β2 (Thr 40-Ser 43), and loop2 (Ser 33-Asp 39). In trimer, loop1 of monomer “a” interacts with the protease domain of monomer “c”, while β1 and β2 of monomer “a” form parallel β-sheets with β2 of monomer “b” and β1 of monomer “c”, respectively (Figure 8). Consequently, the area of interaction between adjacent subunits in the trimer is much larger in *H. pylori* HtrA than in other HtrAs of known structure, where trimers are maintained mainly via hydrophobic interactions between the adjacent protease domains [16].

The aforementioned features of the *H. pylori* HtrA trimer structure allow two main conclusions to be drawn: (1) the N-terminal region is crucial for the maintenance of the oligomer, and (2) the N-terminal domain swapping should provide the oligomer with particular stability. The experimental data confirm both statements. First, the absence of the N-terminal fragment in HtrA results in the loss of ability to form trimers [16,75]. The unstable trimers of N-terminally truncated HtrA reported by [17] may be due to the presence of His tags at the N-termini of recombinant HtrA, which could potentially mediate interactions at the subunit–subunit interface in the trimeric structure. In general, the inability to form a trimer translates directly into a lack of proteolytic activity of HtrA [16,17,75]. Second, *H. pylori* HtrA is characterized by exceptional structural stability and substrate degradation ability over a very wide range of temperature and pH values. Overall thermal stability of this protein does not depend on pH (in the range of 5.5–8.0), and the protein melting point (T_M_) value is significantly higher than that of *E. coli* HtrA (approximately 14 °C higher at pH 6.4) [74]. Thus, it is highly likely that strengthening the inter-subunit interactions by domain swapping contributes to the overall stability of the protein, which may translate into its efficient functioning in the harsh stomach environment.

**Figure 9:**
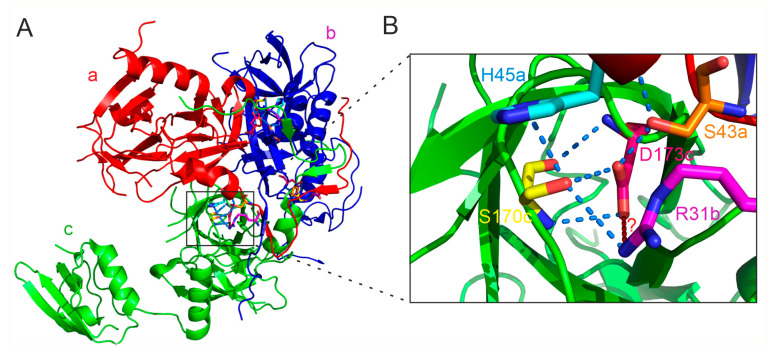
Potential interactions of Asp173 (D173) with amino acid residues located at three monomers “a”, “b”, and “c” are shown on the entire structure (**A**) and fragment in detail (**B**). Hydrogen bonds are shown as blue dashed lines. Potential hydrogen bonds are shown as a red dotted line and question mark. The residues discussed in the text are marked in colors: R31 (magenta), S43 (orange), H45 (cyan), S170 (yellow), and D173 (hot pink). Based on PDB entry 5Y28. The protein structure images were generated using PyMOL version 1.3.

The N-terminal segments are not the sole regions important for maintaining trimeric assembly. In particular, Asp173 plays an important role in stabilizing the trimer. This residue is located at the inter-subunit interface and forms contacts with residues of the neighboring subunits: Ser43 (β2, “c”-subunit) and Arg31 (β1,” b”-subunit) (Figure 9). This inter-subunit connection seems to be crucial for trimerization and protein activity; the HtrA D173A variant is monomeric and devoid of proteolytic activity [16,17]. Asp173 is a component of a long loop (aa 153–173), which connects the N- and C-terminal barrels of the proteolytic domain (see the scheme in Figure 3). Some other residues of this loop are important for trimer stability as well. Specifically, mention should be made of the Asp165, Ser166, Asp168, and Ser/Leu170 residues. Substitutions D165A, S166A, and D168A result in a lower trimer stability under conditions of polyacrylamide electrophoresis; however, the proteolytic activity of the mutant variants is maintained [76].

**Figure 10:**
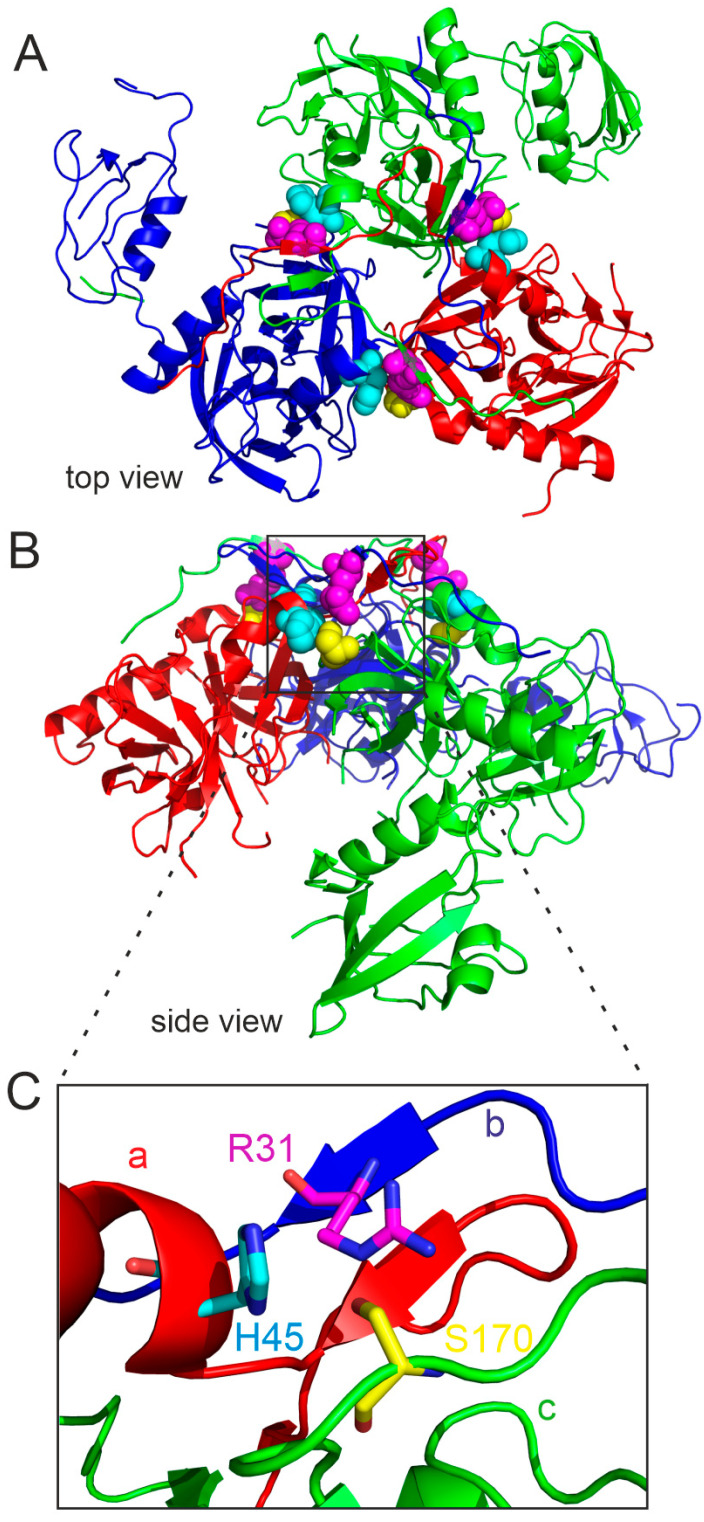
Location of the Ser170 residue at the subunit interface in the HtrA*_Hp_*ΔPDZ2 trimer: (**A**) top view, (**B**) side view, (**C**) enlargement of the contact region of the three subunits “a” (red) “b” (blue), and “c” (green) near the residue 170 (based on PDB entry 5Y28). Positions of Arg 31 (R31), His 45 (H45), and Ser 170 (S170), each of which is located on a different monomer, were marked as (**A**) balls and (**B**) sticks colored in magenta, cyan, and yellow, accordingly. The structure images were generated using PyMOL version 1.3.

In the case of position 170, the presence of Ser lowers the stability of the trimer, while Leu provides higher stability [77] (Figure 10). According to the crystal structure [16], the residues Asp165, Asp 168, and Ser170 form contacts with residues of the adjacent subunits as well. However, these bonds are not as strong as those formed by Asp173, which may explain the weaker effects of the mutations. Ser166 (as well as Ser164) forms contacts with the proteolytic domain α-helix from the same subunit. Hence, in this case the interactions involving these Ser residues may contribute to maintaining the proper spatial structure of the loop. In conclusion, it can be proposed that the interactions formed by the loop components and/or its appropriate structure are necessary to maintain the trimeric structure and thus ensure adequate proteolytic activity of HtrA.

The above observations appear to translate to the function of HtrA in vivo during infection of the host cells by *H. pylori*. A natural single nucleotide polymorphism was observed among *htrA* genes from different *H. pylori* strains, resulting in the presence of Ser or Leu residues at position 170 of the protein. As mentioned above, the HtrA variants S170 and L170 differ in the stability of the trimer. It turned out that the occurrence of the more stable variant correlates with higher efficiency of damaging the junctions between gastric epithelial cells and more efficient delivery of the CagA oncoprotein to the host cells. Interestingly, it was found that infections with strains with the HtrA L170 variant correlated with a higher incidence of malignant neoplastic lesions than with *H. pylori* expressing HtrA S170 [78].

## 6. Proteolytic vs. Chaperone-Like Activity

A feature of many HtrA proteins is the presence of an additional chaperone-like activity. In the case of *E. coli* DegP, a temperature-dependent mechanism for switching between proteolytic and chaperone-like activities has been proposed [46]. According to the presented model, DegP is a chaperone at a low temperature (below 30 °C), and at 37 °C and above, proteolytic activity predominates. However, later publications suggest that the temperature value alone is not a sufficient factor for “switching” between these two activities. DegP in the presence of an appropriate substrate shows significant proteolytic activity even at low temperatures [79]. Moreover, its chaperone-like activity efficiently protects bacterial cells from the effects of thermal stress and limits the accumulation of protein aggregates [34]. The presence of proteolytically inactive HtrA is sufficient to protect against the negative effects of overproduction of proteins of abnormal structure [80]. In many other bacterial species, HtrA proteins are also expected to exhibit dual proteolytic and chaperone activity, but the determinant of the “switch” of these activities is also unknown. Possibly, it is a substrate-dependent issue or, as recently proposed, a matter of filling the sufficient number of binding sites in the oligomeric cage [33].

The chaperone function of HtrA has been the subject of much discussion in the literature and some researchers have questioned the physiological significance of chaperone-like activity of HtrA (e.g., [81]). Nevertheless, the following has been demonstrated:

a Proteolytically inactive HtrAs prevent aggregation of improperly folded proteins. *E. coli* DegP [34], *B. pertussis* DegP [82], *Stenotrophomonas maltophilia* HtrA [83], *C. jejuni* HtrA [67], *H. pylori* HtrA [84], and other homologues can suppress aggregation of model substrates in in vitro tests. Moreover, *E. coli* DegP S210A reduces the level of aggregates in bacterial cells grown under thermal stress conditions [34].
b The presence of proteolytically inactive HtrA variants in bacterial cells abolishes the phenotypes of sensitivity to some stress conditions, including thermal stress, non-physiological pH values, and oxidative stress [67,84,85,86].
c It is assumed that the chaperone activity of HtrA may play a role in the biogenesis of the outer membrane in bacteria—DegP proteins may participate, together with other chaperones, in the delivery of OMPs or autotransporters to the outer membrane [87,88]. On the other hand, there are publications demonstrating that DegP primarily acts as a protease towards developed OMPs in the in vivo system [89]. A recent publication demonstrates that DegP degrades complexes of the Skp (Seventeen kilodalton protein) periplasmic chaperone with OMPs that cannot enter the OM via the BAM system [90].
d There are results indicating that HtrA chaperone activity (but not proteolytic activity) is important in the export/maturation processes of some virulence factors in pathogenic bacteria, for example, IcsA of *Shigella flexneri* [91].

For the reasons listed above, it is important to note that inhibition of HtrA proteolytic activity in pathogenic bacteria may be insufficient to kill the bacteria and prevent the secretion of virulence factors. Therefore, in these particular cases, the possibility of eradication of the microorganism by molecules that inhibit the proteolytic activity of HtrA should be approached with great caution.

## 7. Conclusions

Proper regulation of enzyme activity is of paramount importance for the proper functioning of the organism. The enzyme should perform its function at the right place and time, with the proper intensity and against the correct substrate. The proteins of the HtrA family are a very interesting example of a multilevel mechanism for regulating proteolytic activity to meet the above requirements. In the well-characterized representatives, DegS and DegP proteins from *E. coli* bacterium, modulation of their activity is based on the following strategies:

1. In the absence of activators/substrates, these proteins exist in an inactive resting form, characterized by an inappropriate conformation of the active center.
2. Conversion to the active form of the protease occurs in the presence of appropriate levels of substrate proteins or activating peptides.
3. In the housekeeping DegP protease, there is an additional level of regulation by hiding the active catalytic sites inside the cavity of higher-order oligomers, which further prevents uncontrolled proteolysis.

The above strategies are implemented via changes in HtrA inter-domain contacts and accompanying oligomer rearrangements.

Activation of both proteases by a similar type of ligands (C-terminal peptides that are exclusively exposed by OMP proteins with an abnormal structure) provides simultaneous activation of the σ^E^-dependent response to envelope stress (due to DegS activity) and activation of housekeeping DegP protease, which removes abnormal proteins and prevents their aggregation.

Much less is known about the structures and activity regulation of HtrA homologs from other Gram-negative bacterial species. The relatively high similarity of amino acid sequences and domain structures observed among proteins of this family suggests the presence of similar regulatory mechanisms to those described for homologs from *E. coli*. However, the results of structural studies performed on *C. jejuni* HtrA and *H. pylori* HtrA indicate the presence of important conformational differences from model HtrAs. Therefore, a thorough biochemical characterization of these proteins, especially with respect to allosteric regulation of their activity, is required.
